# The True Relation between the Pharmacist and the Physician*Read before the Hardin County Medical Society.

**Published:** 1898-10

**Authors:** Garrett D. Smock


### THE TRUE RELATION BETWEEN THE PHARMACIST AND THE PHYSICIAN.*

BY GARRETT D. SMOCK, M. D.

Every institution extant in the world to-day has been called into existence to serve the necessities of mankind. The existence of the institution of government itself is due to this cause. The necessity for law and order, for the peaceful dwelling together of mankind, made it necessary that a power should be created to which all would submit. Every other institution when investigated will be found to have originated to serve man's necessities and comforts. Also every institution when investigated will be found to have originated from a chaotic state and to have been evolved gradually from small beginnings to a state of more or less perfection.

Medicine and pharmacy are no exceptions to this rule. We find them existing, after a sort, away back in very remote ages of the world's history, no doubt from the very infancy of the race. Man, from his very nature, physically was created subject to disease and death. The commonly received notion that the eating of the fatal apple by our great progenitor, Adam, first introduced disease and death is, I think, very erroneous. The remedies used to alleviate human suffering in remote ages were necessarily few and simple, and medicine and pharmacy were both in the hands of the same person, most generally the priest. They were, in fact, looked upon as a part of religion, and as the materia medica was very brief, it was supplemented by prayers and incantations and sorcery, or witchcraft. I am sorry to have to say, too, that such modes of practice have not become altogether obsolete in the world at the present day.

Prescription writing seems to have been practiced at a very early day. Thousands of them have been exhumed from the ruins of Babylon, Nineveh, Persepolis, and other Asiatic cities. They were written upon baked clay tablets. Many of them are medical prescriptions conjoined with magical practices, such as the incantations and exorcisms repeated over the sick. Some contain directions for compounding magical drinks which would destroy the evil spirits by which the particular disease was caused. No doubt these drinks were merely antiseptics or germicides intended to destroy the microbes which were then called evil spirits. I have a *fac-simile* of a prescription four thousand years old, and said to be the oldest prescription extant. The original is in the British Museum. It is written in cuniform characters, and although the language in which it is written has been dead for thousands of

*Read before the Hardin County Medical Society.

years, it is about as easy to read as a great many prescriptions which come into the hands of every druggist at the present day.

Both medicine and pharmacy continued in the hands of the same individual, who, as I have said, was generally the priest, until a comparatively recent time. Pharmacists finally in the reign of James the First obtained a charter for themselves apart from the practitioners of medicine, and although their charter included the grocers, the medical profession are in no position to sneer when it is remembered that at the same time barbers were the only recognized surgeons. Both pharmacy and surgery seem to have been at a discount long after they were divorced from the medical profession, and both had a long struggle before they were recognized as professions equally as honorable and as deserving as medicine. Indeed, surgery seems to have outstripped medicine at the present day, for while formerly the most learned and ambitious practiced medicine, the tendency now is to ignore medicine as tame and commonplace, and to rush into surgery. The constant dream of every ambitious young doctor now is to become a great surgeon. The knife is resorted to in every case in which its use can possibly be justified, and I am afraid in a great many where it could not be justified.

The rapid strides made in the science of medicine and surgery as a result of a general revival of learning and original research made it absolutely impossible for physicians and surgeons to compound their remedies, now indefinitely multiplied in numbers, and make them available at the bedside. This work was necessarily entrusted to the pharmacists, and they have nobly responded to the trust. They have established colleges and training schools of their own in every civilized country, where the science of pharmacy is taught as a profession, and scientific pharmacists are trained for their work. They have prosecuted original research until to-day scientific pharmacy stands fully abreast with the science of medicine, and physicians are under many obligations to scientific pharmacy for much of their success in the practice of the healing art. But the pharmacist is not the *rival* of the physician; he is an *ally*. It is in this capacity that the pharmacist finds his true place, and the two their true relationship each to the other. Each is a true helpmeet to the other, working hand in hand, each in his own proper sphere; they each fill a place and work out a destiny equally honorable and equally useful. Neither can work out his highest destiny without the other, for neither can put his talents to the highest and best use without the other. The motto of our beloved Commonwealth was never more forcibly illustrated—"United they stand, divided they fall."

Since, then, the pharmacist and the physician stand in this close relationship the one to the other, there are certain duties which they owe the one to the other and which they should conscientiously discharge. The pharmacist owes it to the physician that he educate himself well in his profession, so that he will be able to compound the prescriptions of the physician in a scientific manner that the patient may derive the full therapeutic effects of medicines prescribed for him. Ignorance is inexcusable anywhere; behind the prescription counter it is criminal. The preparation of his remedies is always a matter of grave moment to the physician, for what avails his skill if it is all to be neutralized by poorly prepared remedies? Too true is it perhaps that ignorance is sometimes found behind the prescription counter, and also too true that it is sometimes found at the bedside, as the prescriptions which sometimes emanate from thence too plainly show; not professional ignorance alone, but the King's English is sometimes treated in a very barbarous manner. The three R's are to some of them still among the mysteries. Gentlemen, whether we be physicians or pharmacists, let us act upon the last words of John Wesley to his followers, "Wherever you go, preach a crusade against ignorance."

It is the duty of the pharmacist also to keep himself well supplied with the remedies usually prescribed by physicians and also the newer remedies, that the physician may have opportunity to test their value and avail himself of their virtues if found beneficial. On the other hand, the physician should not be too exacting in this matter. It is usually the case that one physician will want the article as made by a certain firm, another will want the same article bearing the label of a different firm, and so on. They should remember that to keep a stock of every manufacturer's make of pharmaceutical remedies would entail an expense which but few pharmacists could afford; but should they be able to do so, it would be entirely unnecessary. The article as made by any first-class manufacturer differs but little from that made by any other first-class house, and the physician, if called upon to state wherein the article made by his favorite house differed from that made by another, would often be puzzled to do so. Most generally he prefers it because he happened to fall into the hands of the representative of that particular house, who made large claims to exclusive merit in his preparations because of the exclusive advantages possessed by his house over all others. The only way to possess all the advantages is to possess all the money and all the brains in the business. Any first-class house with first-class business men at its head with ample capital at their command can always secure all the brains and all the material needed to turn out a first-class article.

The pharmacist owes it to the physician also to compound his prescription exactly as he has directed, unless he is satisfied that the physician has made a mistake. In this case he should promptly inform the physician and have the mistake corrected or have the matter fully and satisfactorily explained. Should this pharmacist not have the article prescribed and be unable to obtain it promptly, he should at once inform the physician that he may direct him as to what he prefers having him use in its stead. Should the physician be at the time inaccessible and it be a case of emergency, the pharmacist should simply use his judgment as to the proper thing to be done, only let him be very sure that he is doing the proper thing. Unless it be a case of great emergency he had better wait until the physician can be heard from.

While on this subject, I desire to call your attention to the fact that there is a very bitter warfare being waged at the present time on pharmacists by the proprietors of patent medicines. You may have read articles in the newspapers purporting to be editorials denouncing pharmacists as base and systematic substitutors. These articles breathe a very vicious and malignant spirit, and you may not all know the source from whence they derive their inspiration. When once traced to their true origin, however, their animus is fully explained. There has been for a long time a determination on the part of pharmacists to eliminate the patent medicines from their business. True, scientific pharmacy is as much opposed to patent nostrums as scientific medicine, and pharmacists deprecate the fact that the handling of patent nostrums ever became a part of their business. In fact, pharmacists are opposed to all secrecy in connection with the preparation of pharmaceutical remedies. The American Pharmaceutical Association at its last meeting passed a resolution severely condemning the patenting or trade-marking of any remedy in medicine. A great many pharmacists who are prepared to do so are manufacturing preparations of their own with the formula on the bottle. Others who are not prepared to manufacture are having the work done for them.

The patent medicine men have organized to fight this, and have established a literary bureau from which emanate these vicious attacks upon pharmacists which are published in the various newspapers and other periodicals, for which they pay the papers a big price. These articles are intended to lower the standing of pharmacists and to destroy their customers' confidence in them. The last national meeting of the patent medicine proprietors voted a large sum of money to continue this warfare.

The question is being discussed now in pharmaceutical circles whether or not pharmacists should be graduates in medicine. There exists a difference of opinion, but all are agreed that they should possess considerable knowledge of at least some branches of medical science, as such knowledge would enable them to more fully appreciate the responsibilities of the position which they occupy. It is also desirable that physicians should be taught a certain amount of pharmacy, that they may be able to distinguish between true pharmacy and pharmaceutical quackery, and also that they may be better fitted to practice their own profession successfully when they are compelled to be their own pharmacists.

Before closing this paper I desire to emphasize the importance of physicians writing their prescriptions plainly and not abbreviating too much. Don't leave the pharmacist to guess at the meaning. Deaths have resulted from this. One occurred recently at Covington, Ky. The physician prescribed powd. ipecac, abbreviating it to "ipe," but the writing was so bad that the pharmacist read it "opi," and death was the result. The question now before the courts is, who is to blame?

The physician should always accord to the pharmacist liberal treatment. He should not take advantage of the pharmacist's liberality toward him to deprive him of his legitimate profit on his work and on his drugs. Sometimes the physician will have his own prescriptions compounded, paying the pharmacist little if any above the absolute cost of the crude material, which he then resells to his patient, charging him a large profit thereon, thus reaping not only the reward for his own services, but also that which legitimately belongs to his fellow-laborer, the pharmacist. This is entirely wrong, and fails to show a just and liberal spirit. On the other hand, physicians will sometimes have their own prescriptions compounded, which they will resell to their patients at cost or perhaps charge nothing for them whatever. This is equally wrong, for it not only deprives the pharmacist of his legitimate reward, but also creates dissatisfaction between the pharmacist and these same people when they have to pay for the medicines themselves. Pharmacists will generally sell to physicians at about cost, but they could not afford to do so with the people at large; if they did it would be only a question of time, and in most cases a very short time, when they would have to quit business.

If the pharmacist is making something by reason of your prescriptions, do not begrudge it to him. If he is honest and faithful, he deserves it. If you have a large practice, you are fortunate if you have a good pharmacist in whom you have confidence to prepare your remedies for use at the bedside. I well remember when I was doing a large practice ten miles from any drug store what a task it was to be my own pharmacist. When on arriving at home already very much fatigued and worried I would find several calls waiting, some of them perhaps very urgent, I would also find a number waiting for medicine. I would cheerfully have relinquished all the profit in the medicine, although I always charged a good price for it, could I have had a good pharmacist to whom I could have turned over this part of the business.

Gentlemen, let us all labor for higher attainments, each in his own sphere, and for mutual confidence. Let us each and all strive for the highest proficiency possible for the relief of human suffering. This it is which dist·nguishes a liberal profession from selfish commercialism, and it is only when we are animated by this spirit, with this end in view, that we are worthy of our profession, whether pharmacist or physician.—*The American Practitioner and News.*

